# Exploration of Malignant Characteristics in Neoadjuvant Chemotherapy-Resistant Rectal Cancer, Focusing on Extramural Lesions

**DOI:** 10.1245/s10434-023-13928-z

**Published:** 2023-08-07

**Authors:** Shunsuke Kubota, Takuya Miura, Taiichi Wakiya, Tadashi Yoshizawa, Shintaro Goto, Hajime Morohashi, Yoshiyuki Sakamoto, Yota Tatara, Hiroshi Kijima, Kenichi Hakamada

**Affiliations:** 1 Department of Gastroenterological Surgery, Hirosaki University Graduate School of Medicine, Hirosaki, Aomori Japan; 2 Department of Pathology and Bioscience, Hirosaki University Graduate School of Medicine, Hirosaki, Aomori Japan; 3 Department of Stress Response Science, Center for Advanced Medical Research, Hirosaki University Graduate School of Medicine, Hirosaki, Aomori Japan

**Keywords:** Rectal cancer, Neoadjuvant chemotherapy, Extramural vascular invasion, Tumor deposit, Selenium-binding protein 1

## Abstract

**Background:**

Extramural vascular invasion (EMVI) and tumor deposits (TD) are poor prognostic factors in rectal cancer (RC), especially when resistant to neoadjuvant chemotherapy (NAC). We aimed to define differential expression in NAC responders and non-responders with concomitant EMVI and TD.

**Methods:**

From 52 RC surgical patients, post-NAC resected specimens were extracted, comprising two groups: cases with residual EMVI and TD (NAC-resistant) and cases without (NAC-effective). Proteomic analysis was conducted to define differential protein expression in the two groups. To validate the findings, immunohistochemistry was performed in another cohort that included 58 RC surgical patients. Based on the findings, chemosensitivity and prognosis were compared.

**Results:**

The NAC-resistant group was associated with a lower 3-year disease-free survival rate than the NAC-effective group (*p* = 0.041). Discriminative proteins in the NAC-resistant group were highly associated with the sulfur metabolism pathway. Among these pathway constituents, selenium-binding protein 1 (SELENBP1) expression in the NAC-resistant group decreased to less than one-third of that of the NAC-effective group. Immunohistochemistry in another RC cohort consistently validated the relationship between decreased SELENBP1 and poorer NAC sensitivity, in both pre-NAC biopsy and post-NAC surgery specimens. Furthermore, decrease in SELENBP1 was associated with a lower 3-year disease-free survival rate (*p* = 0.047).

**Conclusions:**

We defined one of the differentially expressed proteins in NAC responders and non-responders, concomitant with EMVI and TD. SELENBP1 was suspected to contribute to NAC resistance and poor prognosis in RC.

**Supplementary Information:**

The online version contains supplementary material available at 10.1245/s10434-023-13928-z.

To improve the prognosis of rectal cancer (RC) patients, controlling not only local recurrence but also distant metastases is required.^1^ Conventionally, the global standard for RC management is total mesorectal excision (TME) aiming for a negative circumferential resection margin. In recent years, the efficacy of neoadjuvant therapy (NAT) followed by TME have been demonstrated.^2,3^ NAT includes neoadjuvant chemoradiotherapy (NACRT), neoadjuvant chemotherapy (NAC), and total neoadjuvant therapy (TNT), which is a combination of both therapies. Although NACRT is recommended in the National Comprehensive Cancer Network’s guidelines,^4^ radiation complications cause bowel dysfunction as well as urinary and sexual dysfunctions.^5^ A randomized controlled trial (RCT) revealed that NAC without radiation had equal oncologic outcomes and provided better anorectal function.^6^ Furthermore, recent RCTs found that the addition of NAC to radiation therapy reduced distant metastases.^7,8^ In this context, the role of NAC is becoming increasingly important. We focused on NAC using oxaliplatin and S-1, an oral 5-fluorouracil drug that has shown efficacy for locally advanced RC (LARC).^9^

Extramural vascular invasion (EMVI) and tumor deposits (TD) are magnetic resonance imaging (MRI) findings in RC (mrEMVI and mrTD), of which the prevalence is 36.0–49.5% and 47.5–51.0%, respectively.^10,11^ These findings are known to be associated with poor prognosis.^10–12^ In particular, NAC non-responders with residual EMVI and TD after NAC (ymrEMVI and ymrTD) have been reported to have higher local and distant recurrence rates even after R0 resection, while NAC responders whose EMVI and TD disappeared after NAC had a good prognosis.^13^ From these findings, identification of the biological characteristics of NAC non-responders that contribute to the principal malignant property is necessary but remains largely undefined.

The aims of this study were to (1) define differential protein expression in NAC responders and non-responders with ymrEMVI and/or ymrTD using proteomic analysis; and (2) validate the findings using immunohistochemistry (IHC). We conducted proteomic analysis and IHC using LARC resected specimens that clinically resisted NAC. In this study, we present novel findings that reflect the chemoresistance and poor prognosis of LARC.

## Methods

### Proteomic Analysis Study

#### Patients

This single-center, retrospective, observational study was approved by the Committee of Medical Ethics of Hirosaki University Graduate School of Medicine (reference no. 2021-120). Informed consent was obtained in the form of an opt-out option (https://www.med.hirosaki-u.ac.jp/hospital/outline/resarch/resarch.html), with the approval of the Committee of Medical Ethics of Hirosaki University Graduate School of Medicine. This study was designed and carried out in accordance with the Declaration of Helsinki.

**Fig. 1:**
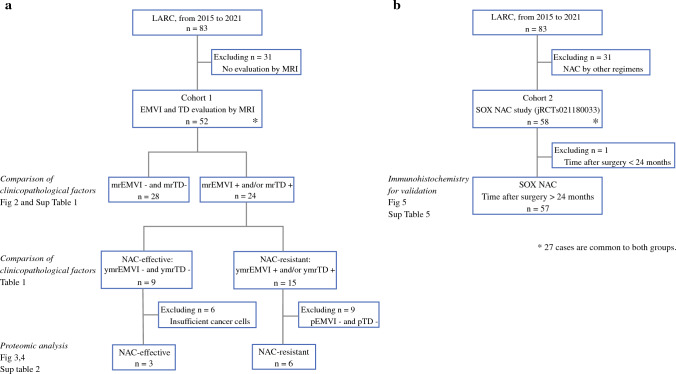
Study design, consisting of two cohorts: **a** Cohort 1 was set up for proteomic analysis; **b** Cohort 2 was set up for IHC for validation. *LARC* locally advanced rectal cancer, *MRI* magnetic resonance imaging, *EMVI* extramural vascular invasion, *TD* tumor deposit, *SOX* S-1 and oxaliplatin, *NAC* neoadjuvant chemotherapy

The study flowchart is shown in Fig. 1. A total of 83 patients undergoing NAC followed by radical surgery for lower RC at our facility from June 2015 to April 2021 were included. All patients had a histologically confirmed adenocarcinoma and the clinical stage was determined with computed tomography (CT) or MRI upon initial examination. Cancers were categorized as cStage II–III based on the classification system of the Union for International Cancer Control (UICC), 8th edition.^14^

**Fig. 2:**
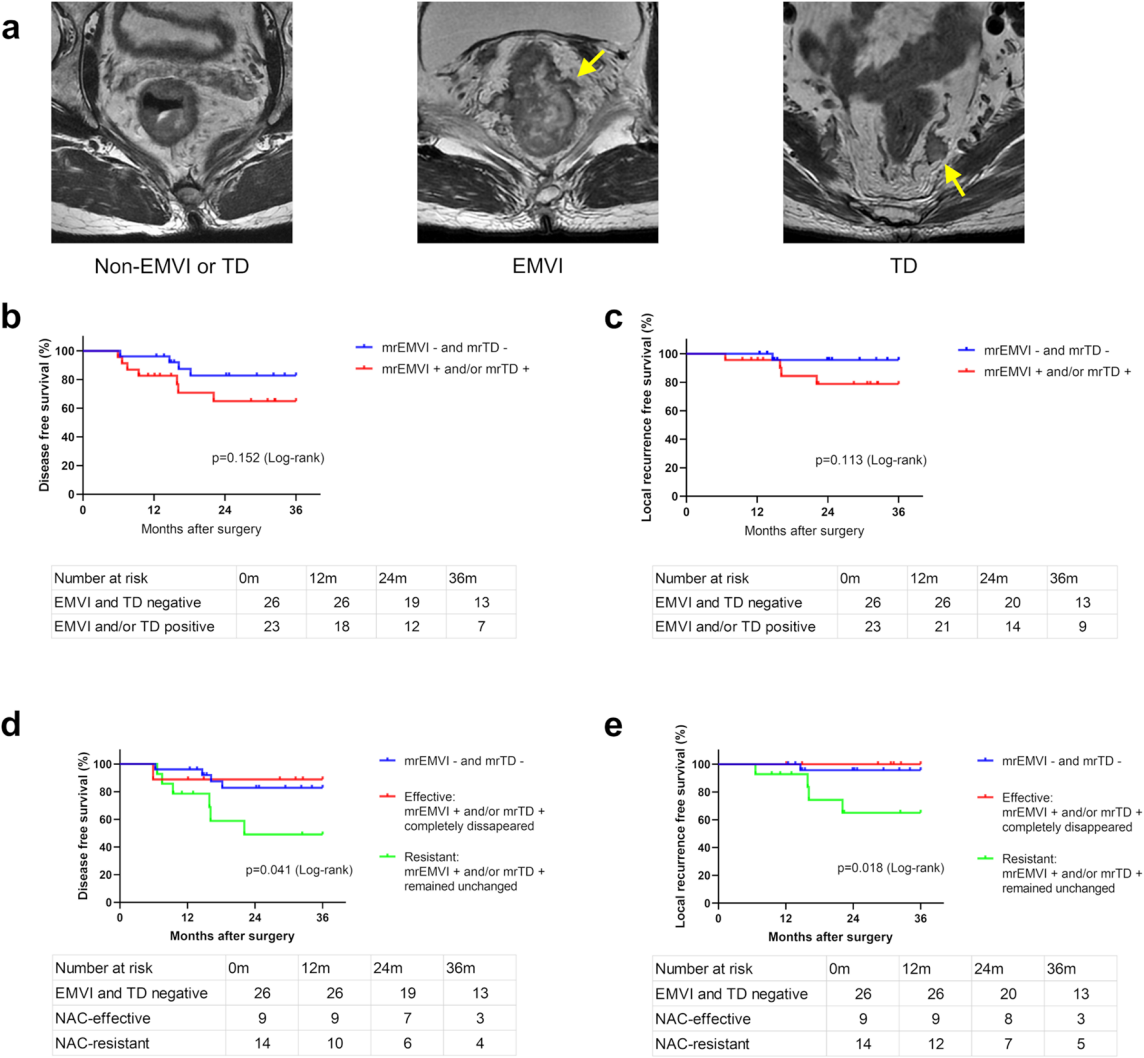
**a** Characteristic findings of EMVI and TD on MRI. EMVI is identified as irregular and expanding vessels (yellow arrow) with definite tumor signals, and TD is identified as irregular nodules in the mesorectal fat separated from the primary tumor, interrupting the course of the vein (yellow arrow). **b**, **c** Disease-free and local recurrence-free survival in the mrEMVI- and mrTD-negative cohort (*n* = 26) and mrEMVI- and/or mrTD-positive cohort (*n* = 23). **d**, **e** Disease-free survival and local recurrence-free survival in the mrEMVI- and mrTD-negative cohort (*n* = 26), the NAC-effective group (*n* = 9) in whom mrEMVI- and/or mrTD-positive completely disappeared, and the NAC-resistant group (*n* = 14) in whom mrEMVI- and/or mrTD-positive remained unchanged. *MRI* magnetic resonance imaging, *EMVI* extramural vascular invasion, *TD* tumor deposit, *NAC* neoadjuvant chemotherapy

During the period from May 2017 to April 2021, 52 patients were selected for a proteomic analysis study (Cohort 1) (Fig. 1a). Patients were evaluated using high-resolution MRI scans directly before and after NAC. EMVI is identified as irregular and expanding vessels with definite tumor signals, and TD is identified as irregular nodules separated from the primary tumor in the mesorectal fat, interrupting the course of the veins (Fig. 2a). Radiological assessment was performed by a board-certified radiologist.

#### Neoadjuvant Chemotherapy (NAC)

The indications for NAC were determined according to the clinical study, registered with the Japan Registry of Clinical Trials (jRCTs021180033 and 021180023). S-1 and oxaliplatin NAC (SOX NAC) was administered for LARC from 2015 to 2020, prior to surgery, in accordance with the criteria of jRCTs021180033. SOX plus bevacizumab NAC (SOX-B NAC) was administered for high-risk LARC, including cT4 and lateral lymph node metastasis, from 2018 to 2021, in accordance with the criteria of jRCTs021180023. SOX NAC included the following: S-1 was administered orally at 80 mg/m^2^/day for 14 consecutive days, followed by a 7-day resting period; oxaliplatin was administered intravenously on the first day at a dose of 130 mg/m^2^/day. The duration of one cycle was 21 days, and the SOX NAC consisted of three cycles. SOX-B NAC included the following: bevacizumab was administered intravenously on the first day of each cycle and the other drugs were administered in the same manner as SOX NAC. The duration of one cycle was 21 days, and SOX-B NAC consisted of four cycles.

#### Surgery

Surgery was carried out 3–8 weeks after NAC ended. The types of operations performed included low anterior resection, intersphincteric resection, and abdominoperineal resection. All patients underwent laparoscopic surgery or robot-assisted laparoscopic surgery.

#### Histological Evaluation

All slides, which were originally prepared from formalin fixed and paraffin-embedded tissue of surgical resected specimens, were reviewed. Morphological analyses were performed using 4 μm slides stained with hematoxylin and eosin (H&E). Pathological EMVI and TD (pEMVI and pTD) were diagnosed based on the Japanese Society for Cancer of the Colon and Rectum standards.^15^ pEMVI was defined as extramural cancer lesions involving vascular invasion, and pTD was defined as non-lymph node lesion extramural discontinuous cancer spread.

#### Immunohistochemistry

The method of immunohistochemical staining is described in the electronic supplementary Methods.

IHC results were evaluated in sections, using the largest tumor surface according to histological score (H-score; range 0–300).^16^ This semiquantitative score was obtained by multiplying the total cytoplasm immunoreactivity intensity (scored as 0, 1, 2, or 3) and corresponding percentage of stained cells (0–100%). The intensity score was defined as follows: 0, no appreciable cytoplasm staining (negative); 1, barely detectable staining in the cytoplasm (weak); 2, readily appreciable brown staining distinctly marking the cytoplasm (moderate); 3, very dark cytoplasm staining (strong). The scores were evaluated by two pathologists.

#### Classification for Proteomic Analysis

Patients who were mrEMVI-positive and/or mrTD-positive on pre-NAC MRI were selected as the proteomic analysis cohort. These patients were divided into two groups according to the presence/lack of ymrEMVI and ymrTD on post-NAC MRI. The classification method was as follows: NAC-effective group, ymrEMVI negative and ymrTD negative; and NAC-resistant group, ymrEMVI-positive and/or ymrTD-positive.

#### Tissue Section Preparation for Mass Spectrometry

See the electronic supplementary Methods.

#### Liquid Chromatography with Tandem Mass Spectrometry (LC–MS/MS) and Proteomic Data Analysis

See the electronic supplementary Methods.

### Validation Study

#### Patients

During the period from June 2015 to January 2020, 58 patients were selected to validate the findings of the proteomic analysis (Cohort 2) (Fig. 1b), of whom 27 patients were recruited from Cohort 1. The data used in this study were collected prospectively through clinical study (jRCTs021180033).

#### NAC

SOX NAC was administered according to the same criteria as Cohort 1.

#### Surgery

Surgeries were carried out according to the same criteria as Cohort 1.

#### Histological Grading Based on the Therapeutic Effect of NAC

Pathological tumor response to NAC, based on the Japanese Society for Cancer of the Colon and Rectum standards,^15^ was classified as follows: Grade 0, no tumor cell necrosis or degeneration in response to treatment is observed; Grade 1a, tumor cell necrosis or degeneration is present in less than one-third of the entire lesion; Grade 1b, tumor cell necrosis, degeneration, and/or a lytic change is present in more than one-third, but less than two-thirds, of the entire lesion; Grade 2, prominent tumor cell necrosis, degeneration, a lytic change, and/or a disappearance is present in more than two-thirds of the entire lesion, but viable tumor cells remain; Grade 3, necrosis or a lytic change, or both, are present throughout the entire lesion and have been replaced by fibrosis, so no viable tumor cells are observed. Pathological assessment was performed by board-certified pathologists.

#### Immunohistochemistry

IHC was performed using the same method as Cohort 1.

#### Statistical Analysis

See the electronic supplementary Methods.

## Results

### Proteomic Analysis Study

#### Comparison of the Clinical Characteristics

First, we investigated the clinical characteristics of the 52 enrolled patients in Cohort 1 who were eligible for high-resolution MRI, focusing on the presence/lack of mrEMVI and/or mrTD. Twenty-eight patients (53.8%) made up the mrEMVI- and mrTD-negative cohort, and 24 patients (46.2%) were included in the mrEMVI- and/or mrTD-positive cohort. The results of a comparison are shown in electronic supplementary Table 1. The mrEMVI- and/or mrTD-positive group presented with higher pretreatment carcinoembryonic antigen (CEA) and the patients were mostly at an advanced pathological stage (*p* < 0.05).

Considering the therapeutic effect of NAC, the mrEMVI- and/or mrTD-positive cohort was subdivided into two groups according to the presence/lack of ymrEMVI and/or ymrTD. The clinical and pathological characteristics of these patients are shown in Table 1. Nine patients (37.5%) were classified as the NAC-effective group, whose ymrEMVI and ymrTD were negative. Fifteen patients (62.5%) were included in the NAC-resistant group, whose ymrEMVI and/or ymrTD were positive. The NAC-resistant patients were at an advanced pathological stage and displayed local invasive factors (*p* < 0.005).

**Table 1:** Comparison of the clinicopathological characteristics of the patients

Characteristics	mrEMVI- and/or mrTD-positive [*n* = 24]	NAC-effective [*n* = 9]	NAC-resistant [*n* = 15]	*p*-Value
*Sex*				
Male [*n* (%)]	19 (79.2)	9 (100.0)	10 (66.7)	0.071
Age, years	64 (34–78)	65 (57–78)	62 (34–73)	0.591
BMI, kg/m^2^	21.1 (18.3–28.9)	20.7 (18.3–28.4)	21.5 (18.6–28.9)	0.698
CEA, ng/mL	6.2 (0.9–115)	4.2 (0.9–36.2)	8.8 (2.8–115)	0.121
*UICC 8th edition [n (%)]*				
T category				0.418
cT3/T4	14 (58.3)/10 (41.7)	6 (66.7)/3 (33.3)	8 (53.3)/7 (46.7)	
N category				0.640
cN0/N1-2	5 (20.8)/19 (79.2)	2 (22.2)/7 (77.8)	3 (20.0)/12 (80.0)	
Mesorectal LN	18 (75.0)	7 (77.8)	11 (73.3)	0.603
Lateral LN	11 (45.8)	3 (33.3)	4 (26.7)	0.300
*MRI factor [n (%)]*				
EMVI	24 (100.0)	9 (100.0)	15 (100.0)	
TD	11 (45.8)	4 (44.4)	7 (46.7)	0.625
MRF involvement	13 (54.2)	4 (44.4)	9 (60.0)	0.375
NAC [*n* (%)]				0.500
SOX/ SOX + bevacizumab	12 (50.0) /12 (50.0)	5 (55.6) /4 (44.4)	7 (46.7)/8 (53.3)	
NAC completion rate	22 (91.7)	8 (88.9)	14 (93.3)	0.620
Down staging	10 (41.7)	6 (66.7)	4 (26.7)	0.067
Surgical procedure, *n*				0.221
LAR/ISR/APR	11 (45.8)/6 (25.0)/7 (29.2)	6 (66.7)/2 (22.2)/1 (11.1)	5 (33.3)/4 (26.7)/6 (40.0)	
Tumor size, mm	38 (5–80)	30 (5–65)	40 (25–80)	0.099
*UICC 8th edition*				
pT category [*n* (%)]				< 0.001
pT0-2/T3-4	6 (25.0)/18 (75.0)	6 (66.7)/3 (33.3)	0/15 (100)	
pN category [*n* (%)]				0.067
pN0/N1-2	10 (41.7)/14 (58.3)	6 (66.7)/3 (33.3)	4 (26.7)/11 (73.3)	
fStage [*n *(%)]				0.015
0/I/II/III	3 (12.5)/2 (8.3)/5 (20.8)/14 (58.3)	3 (33.3)/2 (22.2)/1 (11.1)/3 (33.3)	0/0/2 (13.3)/13 (86.7)	
*Histology [n (%)]*				0.04
Tubular adenocarcinoma, well-differentiated type	7 (29.2)	4 (44.4)	3 (20.0)	
Tubular adenocarcinoma, moderately differentiated type	15 (62.5)	3 (33.3)	12 (80.0)	
NA	2 (8.3)	2 (22.2)	0	
*Local invasive factor [n (%)]*				
Lymphatic invasion	7 (29.2)	2 (22.2)	5 (33.3)	0.461
Vascular invasion	18 (75.0)	4 (44,4)	14 (93.3)	0.015
Perineural invasion	19 (79.2)	4 (44.4)	15 (100.0)	0.003
pExtramural vascular invasion	12 (50.0)	0	12 (80.0)	< 0.001
pExtramural tumor nodule	7 (29.2)	0	7 (46.7)	0.019
R0 resection [*n* (%)]	23 (95.8)	9 (100.0)	14 (93.3)	0.625

*EVMI* extramural vascular invasion, *TD* tumor deposits, *NAC* neoadjuvant chemotherapy, *BMI* body mass index, *CEA* carcinoembryonic antigen, *UICC* Union for International Cancer Control, *MRI* magnetic resonance imaging, *LN* lymph nodes, *NA* not available

#### NAC-Resistant ymrEMVI- and/or ymrTD-Positive Patients had Worse Prognoses

We investigated the negative impact of mrEMVI and mrTD on prognosis. Survival analysis was performed excluding three patients who underwent R1 resection. There was no significant difference in the postoperative outcomes between the mrEMVI- and mrTD-negative patients and the mrEMVI- and/or mrTD-positive patients (Fis. 2b, c). However, when classifying the patients based on ymrEMVI and ymrTD, the prognostic implications became obvious. The NAC-resistant group of ymrEMVI- and/or ymrTD-positive patients was associated with a lower 3-year disease-free survival (DFS) rate than the NAC-effective group of ymrEMVI- and ymrTD-negative patients (*p* = 0.041) (Fig. 2d). Moreover, the difference in the locoregional relapse-free survival (LRFS) rate was particularly significant (*p* = 0.018) (Fig. 2e), which indicated that EMVI and TD were bad prognostic factors, especially when resistant to NAC.

#### The Samples for Proteomic Analysis were Extracted from the NAC-Effective and -Resistant Groups

To define the differential expression in the NAC-resistant and NAC-effective groups, we took a proteomic approach using surgically resected RC specimens. Patients who were categorized as pT0 were excluded because the tumor volume was insufficient for liquid chromatography tandem mass spectrometry (LC-MS/MS). To make an accurate comparison, we excluded patients who had no pEMVI or pTD. Finally, three and six RC specimens were selected from the NAC-effective and NAC-resistant groups, respectively. The characteristics of the patients are shown in electronic supplementary Table 2.

#### Proteomic Profiling of NAC-Resistant Rectal Cancer

**Fig. 3:**
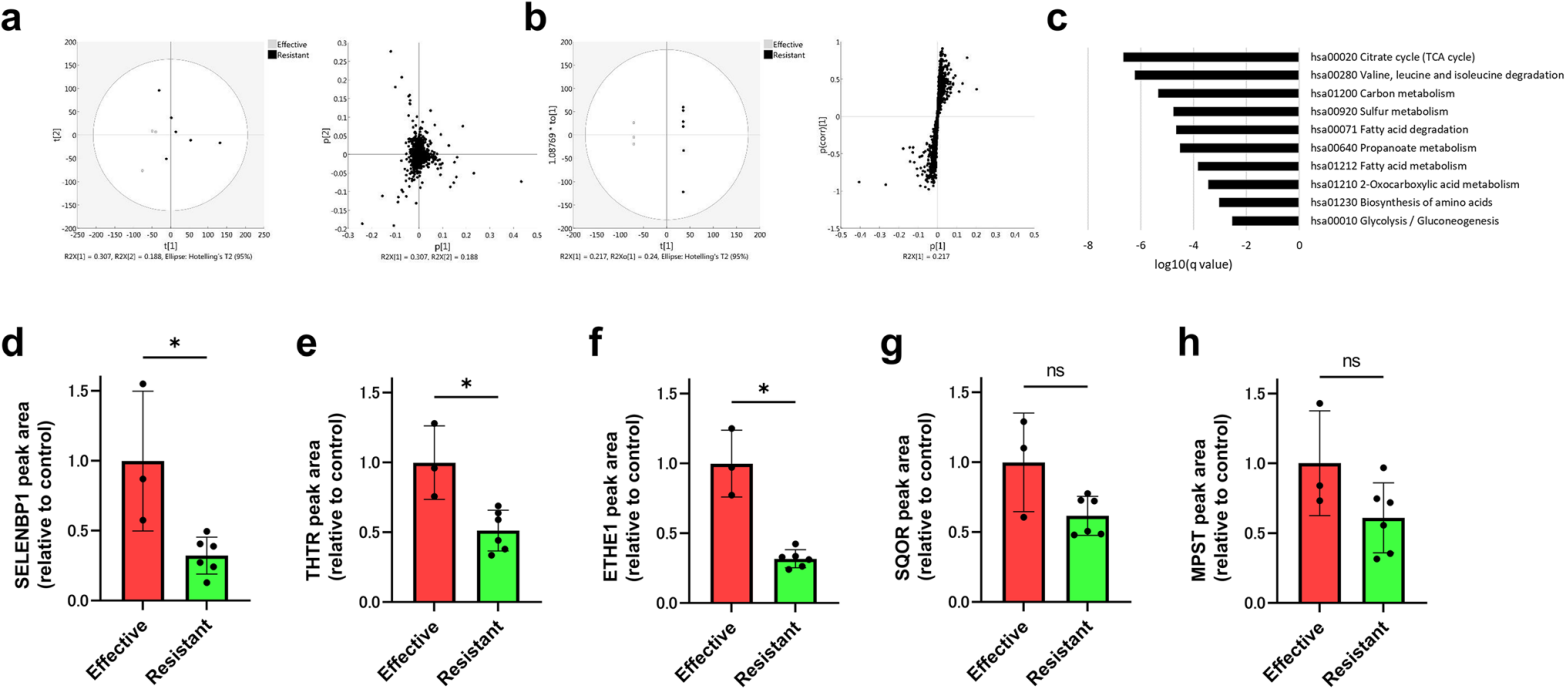
**a** PCA of proteome data (1331 features) from nine samples. The score scatterplot and loading plot are shown in the left and right panels, respectively. **b** OPLS-DA analysis of the proteome data. The score scatterplot and S-plot are shown in the left and right panels, respectively. The vertical axis p1(corr) of the S-plot represents the correlation of each feature to the discriminant model built, with proteins closer to 1 increasing in the NAC-resistant group and proteins closer to − 1 decreasing in the NAC-resistant group. The horizontal axis p1 represents the ratio of change; the higher the value, the more the protein upregulation occurred in the NAC-resistant group. **c** Enrichment analysis of changed proteins using the KEGG pathway. Pathway enrichment analysis was performed on 207 proteins with absolute values of p1(corr) higher than 0.6. The top 10 pathways with enrichment identified in the KEGG pathways are shown. **d–h** Bar plot of relative quantitative values for SELENBP1. The vertical axis represents the normalized relative quantification value output from the MS data using DIA-NN software. Statistical significance was determined using the Mann–Whitney U test. *NAC* neoadjuvant chemotherapy, *KEGG* Kyoto Encyclopedia of Genes and Genomes, *SELENBP1* selenium-binding protein 1, *THTR* thiosulfate sulfur transferase, *ETHE1* sulfur dioxygenase, *SQOR* sulfide quinone oxidoreductase, *MPST* mercaptopyruvate sulfurtransferase, *p < 0.05

Proteomic data analysis was performed comparing the NAC-effective and -resistant groups. First, to provide an overview of the proteome data, the entire dataset, consisting of nine samples and 1331 features, was analyzed by principal component analysis (PCA). As shown in Fig. 3a, there were no major outliers, and the plots of the first and second principal component scores accounted for 30.7% and 18.8% of the original variation, respectively. There was little evidence of discrimination between the two classes along the first and second principal components, suggesting that much of the spectral variation is not related to class differences. Next, we applied OPLS-DA to the proteome data to visualize class separation. The OPLS-DA model concentrates all discriminative information in a first component. The score scatterplot of the OPLS-DA model showed adequate separation between the NAC-effective and NAC-resistant groups using one predictive component and one orthogonal component (Fig. 3b). The results indicate that proteome profiles can be used to distinguish the groups. The resultant S-plot of the developed OPLS-DA model identified 207 proteins with an absolute value of p1, which indicates the correlation with the discriminant model was over 0.6.

#### Selenium-Binding Protein 1 (SELENBP1), a Constituent of Sulfur Metabolism, Decreased in the NAC-Resistant Group

Figure 3c shows the top enriched metabolic pathways with a *q*-value less than −2. Citrate cycle, valine, leucine and isoleucine degradation, carbon metabolism and sulfur metabolism were enhanced, and are involved in energy production in cancer cells. The top 10 enriched pathways and their constituents are listed in electronic supplementary Table 3. We focused on sulfur metabolism; the pathway overview of sulfur metabolism is shown in electronic supplementary Fig. 1. The results of OPLS-DA on five proteins constituting the pathway are presented in electronic supplementary Table 4. Comparing their relative quantitative values, no difference was observed in the expression of eukaryotic sulfide quinone oxidoreductase (SQOR) and mercaptopyruvate sulfurtransferase (MPST). On the other hand, selenium-binding protein 1 (SELENBP1), thiosulfate sulfur transferase (THTR), and sulfur dioxygenase (ETHE1) had significantly decreased in the NAC-resistant group, with a *t*-test *p*-value of 0.02 (Fig. 3d–h). In the NAC-resistant group, SELENBP1 expression was reduced to less than one-third of that in the NAC-effective group.

#### SELENBP1 Decreased in the NAC-Resistant Group Common to the Main Tumor and Extramural Vascular Invasion/Tumor Deposits (EMVI/TD)

**Fig. 4:**
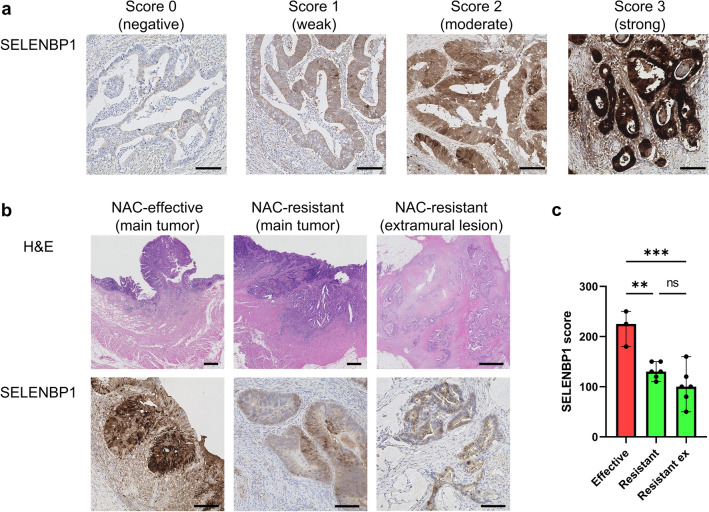
**a** Classification by SELENBP1 staining intensity. **b** Representative immunohistochemistry images of SELENBP1 of resected RC tissues. The NAC-resistant group showed a significantly decreased SELENBP1 expression at the tumor invasion area and the extramural lesions compared with the NAC-effective group. Scale bar = 100 μm. **c** Stainability of SELENBP1 was evaluated and expressed as scores. Statistical significance was determined using the Mann–Whitney U test. *SELENBP1* selenium-binding protein 1, *RC* rectal cancer, *NAC* neoadjuvant chemotherapy, *ns* non-significant, ** *p* < 0.01, *** *p* < 0.001

To investigate the expression of SELENBP1 using a histopathological approach, we conducted IHC using surgically resected RC specimens. IHC for SELENBP1 revealed strong cytoplasmic expression of normal epithelium cells as the positive control. Cancer cells also presented cytoplasmic expression, but the intensity of staining was weaker than that of normal epithelial cells. IHC showed heterogeneity of SELENBP1 expression within tumors, but there was no certain trend in difference between the center and margin of the tumor. We evaluated SELENBP1 expression using the H-score, considering the heterogeneity. We classified SELENBP1 staining intensity into four levels (scores 0–3) (Fig. 4a), counted the proportion of each intensity, and calculated an H-score. The NAC-effective group showed moderate or strong expression, whereas the NAC-resistant group showed weak expression in each of the main tumor and extramural lesions with pEMVI or pTD (Fig. 4b). It was statistically confirmed that the SELENBP1 score was significantly decreased in the NAC-resistant group, in each of the main tumors, its pEMVI and pTD (Fig. 4c).

### Validation Study

#### Evaluation of the NAC Therapeutic Effect in Another Cohort

**Fig. 5:**
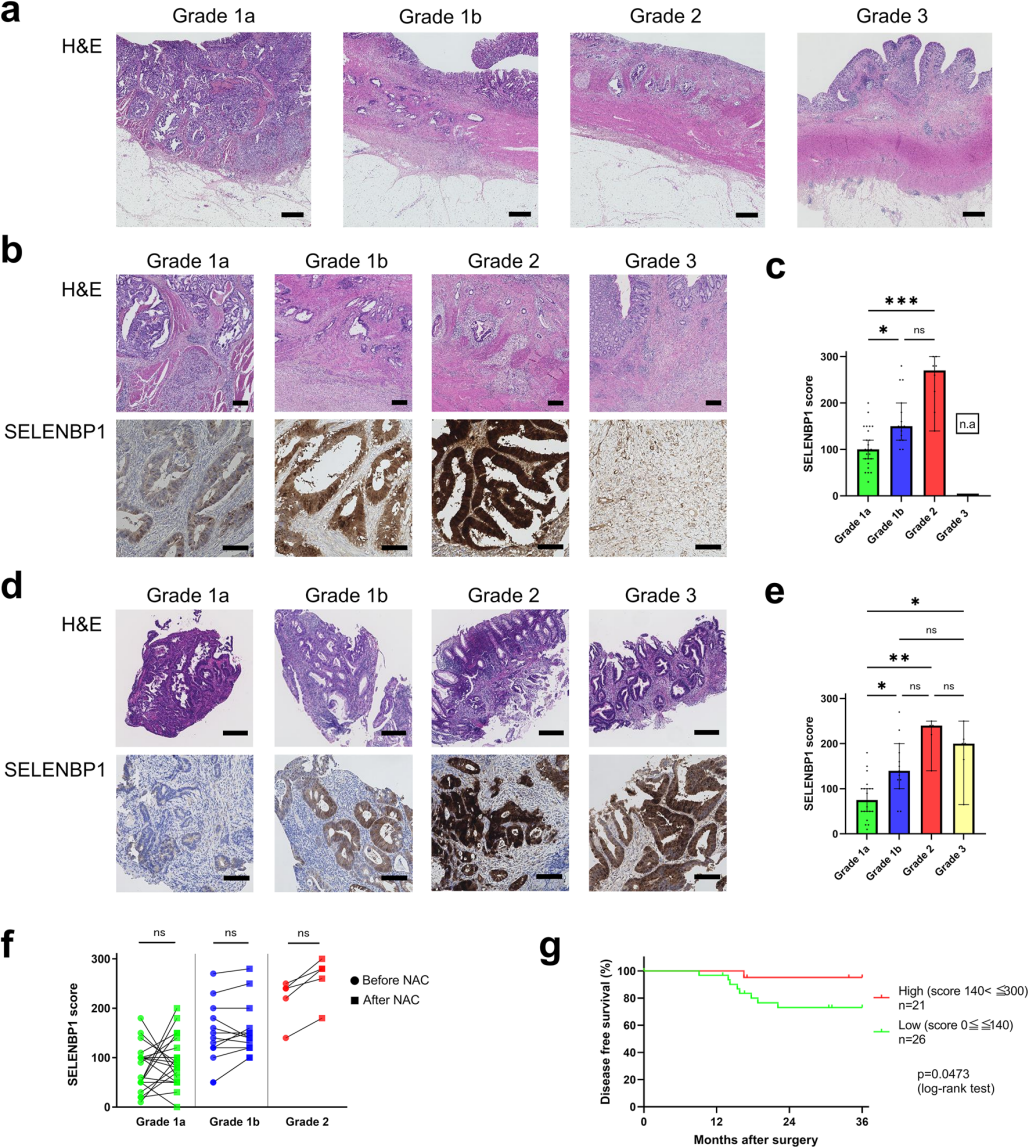
**a** Classification by histological NAC therapeutic grade. Representative images of H&E staining that contribute to the determination of the NAC therapeutic grade. **b** Top: representative images of H&E staining of the surgical specimens resected after NAC. Scale bar = 300 μm. Bottom: representative immunohistochemistry images of SELENBP1 staining. Scale bar = 100 μm. **c** Stainability of SELENBP1 in surgically resected specimens was evaluated and expressed as scores. Statistical significance was assessed using the Kruskal–Wallis test. *p < 0.05, ** *p* < 0.01, *** *p* < 0.001. **d** Top: representative images of H&E staining of pre-NAC biopsy specimens. Scale bar = 300 μm. Bottom: representative immunohistochemistry images of SELENBP1 of biopsy specimens. Scale bar = 100 μm. **e** Stainability of SELENBP1 in biopsy specimens was evaluated and expressed as scores. Statistical significance was assessed using the Kruskal–Wallis test. * *p* < 0.05, ** *p* < 0.01. **f** Comparison of SELENBP1 scores between pre-NAC biopsy specimens and post-NAC surgically resected specimens. Statistical significance was assessed using the Wilcoxon test (*p* < 0.05). **g** Disease-free survival in RC patients with low (*n* = 26) and high (*n* = 21) SELENBP1 scores. The patients were divided into two groups, with a median score of 140 set as the boundary. Statistical significance was assessed using the log-rank test (*p* < 0.05). *NAC* neoadjuvant chemotherapy, *H&E* hematoxylin and eosin, *SELENBP1* selenium-binding protein 1, *RC* rectal cancer, *ns* non-significant

To validate the decrease in SELENBP1 in NAC-resistant RC, we prepared Cohort 2 (Fig. 1b), treated with SOX NAC followed by surgery. All of the 58 patients underwent three courses of SOX NAC followed by radical surgery. One patient whose follow-up period was <24 months was excluded. We evaluated the therapeutic effect of NAC in the section of the largest tumor surface (Fig. 5a). The classification and number of patients was as follows: Grade 1a, 25 patients (43.9%); Grade 1b, 15 patients (26.3%); Grade 2, eight patients (14.0%); and Grade 3, nine patients (15.8%). The clinical and pathological characteristics are shown in electronic supplementary Table 5.

#### Decrease in SELENBP1 was Consistently Associated with Poor Therapeutic Effect in Pre- and Post-NAC Specimens

We assessed the relationship between SELENBP1 expression and NAC therapeutic effect. As with IHC in Cohort 1, SELENBP1 expression was heterogeneous within tumors. IHC using a resected specimen showed a decrease in SELENBP1 in the Grade 1a group, whereas the Grade1b and Grade 2 groups had moderate or strong expression (Fig. 5b). The results were confirmed using the H-score (Fig. 5c). We additionally performed IHC using pre-NAC biopsy specimens. The Grade 1a group presented with lower SELENBP1 scores than the Grade 1b or higher groups (Fig. 5d, e). A decrease in SELENBP1 was associated with an NAC therapeutic effect, which was consistently observed in pre-NAC biopsies and post-NAC resected specimens. These results indicated that the biopsy specimens sampled from the tumor surface could reflect SELENBP1 expression throughout the entire tumor.

#### Pre-NAC Specimens were Comparable with Post-NAC Specimens Despite the Impact of NAC

Next, we compared pre- and post-NAC SELENBP1 scores for each patient. There were no specific trends in changes of SELENBP1 scores for any therapeutic grade group (Fig. 5f). However, focusing on the median scores of each Grade, Grade 1a patient scores averaged out at around 80, while Grade 1b and Grade 2 patient scores averaged out at approximately two- and threefold higher than Grade 1a, respectively. This indicated that pre-NAC biopsy specimens had comparable properties to post-NAC resected specimens with respect to SELENBP1 expression.

#### Decrease in SELENBP1 was Associated with Poor Prognosis of Locally Advanced Rectal Cancer

Finally, to assess the validity of SELENBP1 as a prognostic factor, we performed a survival analysis in Cohort 2. Nine patients with complete response to NAC were excluded because their SELENBP1 expression in resected tissues was not assessable; one patient who underwent R1 resection was also excluded. Consequently, 47 patients were included in the survival analysis. We classified these patients into two groups, with a median SELENBP1 score of 140. As a result of the survival analysis, a lower SELENBP1 score was significantly associated with a lower 3-year DFS rate (*p* = 0.0473) (Fig. 5g).

## Discussion

We revealed that a decrease in SELENBP1 expression is one of the distinctive biological changes in NAC-resistant RC with ymrEMVI and/or ymrTD. Furthermore, we validated this finding and demonstrated that SELENBP1 was related to chemoresistance and lower 3-year DFS in another cohort.

As a result of pathway analysis, we focused on sulfur metabolism. Cysteine and methionine metabolism, types of sulfur amino acids, were related to the cellular antioxidant system, cellular signal transduction, and epigenetic regulation of gene expression. Abnormality in this pathway is known to contribute to malignancy.^17–20^ SELENBP1 was directly linked to cysteine and methionine metabolism. The p1 value of SELENBP1 in OPLS-DA was ranked second in this pathway, indicating that the degree of change was significant. Based on these results, we determined that SELENBP1 deserves further scrutiny.

SELENBP1 has roles in cellular redox modulation, cellular differentiation, metabolism of sulfur-containing protein, and intra-Golgi transport,^21^ and its expression is notably less in human cancer tissue compared with adjacent normal tissue. For years, SELENBP1 has been in the spotlight as a tumor suppressor protein.^21–25^ In human cancer tissue, decreased SELENBP1 expression is correlated with poor prognosis in several types of cancer, including lung,^25^ prostate,^23^ and colorectal carcinoma.^26^ These reports support our findings in the present study. The novelty of our study is targeting RC tissues clinically treated with NAC. This is the first study to demonstrate that decreased SELENBP1 expression is clinically associated with chemoresistance and poor prognosis.

In a validation study, it was indicated that the biopsy specimens sampled from tumor surface could reflect SELENBP1 expression throughout the entire tumor, despite the heterogeneity within tumors. This finding suggested the usefulness of SELENBP1 as a predictive marker for NAC resistance that can be assessed on biopsy. On the other hand, we assessed the effect of NAC on SELENBP1 expression by comparing pre-NAC biopsy and post-NAC resected specimens. As a result, the change in score was not significant. The important finding was that the score of each group remained within an equivalent range. This indicated that a decrease in SELENBP1 can be an inherent property of NAC-resistant RC rather than an extrinsic change caused by NAC. This finding was not completely consistent with previous spheroid studies, but similarly suggested the potential of SELENBP1 as a biomarker for 5-fluorouracil resistance.^27^

TNT has attracted much attention in recent years.^7,8,28^ Since patients with NACRT-resistant EMVI and TD have extremely poor prognosis,^11^ expectations for consolidation or induction chemotherapy are rising. At the same time, studies have been conducted to predict the response to NAT in RC,^29,30^ but none has been applied in clinical situations. Ineffective chemotherapy can cause tumor growth during the NAT period and disturb a radical resection with a sufficient circumferential margin, which is essential to prevent postoperative local recurrence.^31,32^ To evaluate SELENBP1 expression before treatment may contribute to screening out patients who will respond poorly to 5-fluorouracil + oxaliplatin chemotherapy and support the choice of other regimens. This prediction can help to avoid surgical delays and adverse events caused by ineffective chemotherapy; this is beneficial, especially in decisions regarding consolidation or induction chemotherapy.

The present study has several limitations. First, we could not target patients treated with NACRT or TNT. However, since NACRT and TNT include two types of treatment, i.e. chemotherapy and radiotherapy, it would be difficult to discuss the pure relationship between the identified molecules and either one of the treatments. Although NAC is not a standard strategy, the design of this study allowed us to identify a molecule associated with pure resistance to chemotherapy. Since chemotherapy still plays a significant role in NACRT and TNT, we can interpret our results as one step closer to a strong definition of the NAT-resistant factor, not limited to NAC. In Japan, NACRT was not strongly recommended in the period of this study. However, it is now recommended, and our institution has already begun an NACRT strategy. As a prospect for the future, proteomic analysis targeting NACRT cases and comparison with NAC cases would be valuable. Second, this study included only patients treated using the SOX or SOX-B regimens. SOX therapy was shown to be comparable with CapeOX in advanced colorectal cancer.^33^ As for the SOX-B regimen, non-inferiority to mFOLFOX6 + bevacizumab was also demonstrated in advanced colorectal cancer.^34^ We therefore believe our findings in the present study can be applied to global standard therapy. Finally, we performed a proteomic analysis using only the invasive margin of the tumor because we considered that it best reflected the tumor malignancy. However, protein expression patterns can vary depending on the location, even within the same tumor. More predictive or treatable factors could be identified by additional analysis using pretreatment biopsy samples. Since this requires a larger volume of samples than what can be obtained from conventional biopsies, another prospective study is needed.

## Conclusion

We defined one of the differentially expressed proteins in NAC responders and non-responders, with concomitant EMVI and TD. The finding was validated and a decrease in SELENBP1 expression was suspected to contribute to NAC resistance and poor prognosis in RC. SELENBP1 has the potential to be a novel biomarker reflecting chemoresistance in RC and can also assist in decisions regarding NAT.

### Supplementary Information

Below is the link to the electronic supplementary material.

Supplementary file1 (DOCX 24 kb)

Supplementary file2 (DOCX 38 kb)

Figure 1. In the KEGG metabolic pathway map for sulfur metabolism (hsa00920), where rectangles and circles represent enzymes and chemical compounds (substrate and products), respectively. EC numbers and gene names are displayed. Molecules with negative (green) fold change represent molecules that were downregulated in the NAC-resistant group. SELENBP1 (1.8.3.4) is directly associated with cysteine and methionine metabolism. (TIF 5294 kb)

## Data Availability

The proteomic data are available online using access number ‘PXD036382’’ for the ProteomeXchange, and access number ‘JPST001832’’ for the jPOST Repository.
