# Supplementary material for: Exploration of Malignant Characteristics in Neoadjuvant Chemotherapy-Resistant Rectal Cancer, Focusing on Extramural Lesions
Source: Ann Surg Oncol. 2023 Aug 7;30(12):7612–23. doi: 10.1245/s10434-023-13928-z (PMC10562322; doi:10.1245/s10434-023-13928-z)
Supplement: Supplementary file 1 — Supplementary file1 (DOCX 24 kb) [file 10434_2023_13928_MOESM1_ESM.docx]

**SUPPLEMENTAL METHODS**

*Immunohistochemistry*

IHC was performed on formalin-fixed, paraffin-embedded tissue sections of 4μm thickness using standard techniques with a Ventana Benchmark HX Autostainer (Ventana Medical Systems, Tucson, AZ, USA). Antigen retrieval was carried out using Cell Conditioning Solution (CC1, Tris-based EDTA buffer, pH 8.0; Ventana). Following antigen retrieval, tissue samples were incubated for 32 min at 37˚C with rabbit polyclonal anti-selenium binding protein 1/SBP antibody (ab90135, Abcam; 1:1000 dilution). An iVIEW Universal DAB Detection Kit (Ventana) was used for detecting primary antibodies. Positive controls were obtained from normal epithelium distant from the cancer lesion. Images were obtained with an all-in-one microscope (BZ-X700, Keyence) and analyzed with BZ-X Analyzer (Keyence) software.

*Tissue section preparation for mass spectrometry*

Formalin fixed and paraffin-embedded tissues of resected specimens were used for proteomic analysis. Based on the findings on H&E staining, we selected sections from the invasive area of the main tumor and cut out manually. Tissues’ weights ranged 10–20mg.

*Liquid chromatography with tandem mass spectrometry (LC–MS/MS) and proteomic data analysis*

Formalin-fixed, paraffin-embedded tissue was deparaffinized twice with xylene and washed with a descending concentration of ethanol. Proteins in the deparaffinized tissues were extracted in 40 mM Tris-HCl (pH 8.8) containing 0.1% SDS and 10 mM DTT and incubated at 100°C for 20 min and 80°C for 2 h with shaking. Free cysteine residues were alkylated with 20 mM iodoacetamide for 60 min at room temperature in the dark and the remaining iodoacetamide was quenched by adding 10 mM DTT. The mixture was then diluted 10-fold with 100 mM ammonium bicarbonate, and incubated with 1 μg trypsin (TPCK treated, AB Sciex) at 37 °C for 18 h. The samples were desalted with MonoSpin C18 (GL Sciences) and were analyzed by LC-MS using a nanoLC Eksigent 400 system (Eksigent, AB Sciex), coupled online to an TripleTOF6600 mass spectrometer (AB Sciex). Peptide separation was performed using LC on a nano C18 reverse-phase capillary tip column (75 μm × 125 mm, 3 μm, Nikkyo Technos CO).

A DIA-NN software was used to extract peptide signals from raw files using a human protein FASTA sequence database of Swiss-Prot. Raw files of wiff file format were converted to .dia format using the utility embedded in DIA-NN. The following processing parameters were used: a maximum of one missed cleavage and cysteine carbamidomethylation as the fixed modification. FDR was set to 1% using the target-decoy strategy on both the peptide and protein levels.

Statistics of principal component analysis (PCA) and orthogonal partial least square-discriminant analysis (OPLS-DA) were performed using Simca software (Infocom Corp). Pareto scaling was applied to the normalized peak area values acquired by DIA-NN prior to the analyses. Metabolic pathway enrichment analysis of the responsive proteins was conducted according to the information from the KEGG Pathway Database with clusterProfiler by R software.

*Statistical analysis*

Continuous variables were expressed as the medians (ranges) and analyzed using nonparametric methods for nonnormally distributed data: Mann–Whitney U test, Wilcoxon signed-rank test or paired t-test, as appropriate. Categorical variables were reported as numbers (percentages) and analyzed using the Chi-squared test or Fisher’s exact test, as appropriate. Disease free survival (DFS) and local recurrence-free survival (LRFS) were calculated using the Kaplan–Meier method, and differences in the survival rates among the groups were compared using the log-rank test. DFS and LRFS were defined as the time from the operation to the date of recurrence and local recurrence, respectively. This study was planned with a minimum follow-up period of two years. A difference was considered to be significant for values of p < 0.05. The statistical analyses were performed using IBM SPSS Statistics for Windows, Version 26.0 (IBM Corp, Armonk, NY, USA).
