# Supplementary material for: Exploration of Malignant Characteristics in Neoadjuvant Chemotherapy-Resistant Rectal Cancer, Focusing on Extramural Lesions
Source: Ann Surg Oncol. 2023 Aug 7;30(12):7612–23. doi: 10.1245/s10434-023-13928-z (PMC10562322; doi:10.1245/s10434-023-13928-z)
Supplement: Supplementary file 2 — Supplementary file2 (DOCX 38 kb) [file 10434_2023_13928_MOESM2_ESM.docx]

Sup Table 1: Comparison of the clinicopathological characteristics of the patients

| Characteristics | | All (n=52) | | mrEMVI and mrTD negative (n=28) | | mrEMVI and/or mrTD positive (n=24) | | p value | |
| --- | --- | --- | --- | --- | --- | --- | --- | --- | --- |
| Gender | |  | |  | |  | |  | |
| Male, n | | 43 (82.7) | | 24 (85.7) | | 19 (79.2) | | 0.398 | |
| Age, year | | 62 (34-78) | | 61 (36-75) | | 64 (34-78) | | 0.435 | |
| BMI, kg/m^2^ | | 21.4 (15.4-28.9) | | 22.5 (15.4-28.0) | | 21.1 (18.3-28.9) | | 0.898 | |
| CEA, ng/ml | | 4.4 (0.8-148) | | 3.6 (0.8-148) | | 6.2 (0.9-115) | | 0.043 | |
| UICC 8th edition | |  | |  | |  | |  | |
| T category, n | |  | |  | |  | | 0.182 | |
| cT1-2 /T3-4 | | 2 (3,8) /50 (96.2) | | 2 (7.2) /26 (92.8) | | 0 /24 (100) | |  | |
| N category, n | |  | |  | |  | | 0.958 | |
| cN0/N1-2 | | 11 (21.2)/41 (78.8) | | 6 (21.4)/22 (78.6) | | 5 (20.8)/19 (79.2) | |  | |
| Mesorectal LN | | 8 (73.1) | | 20 (71.4) | | 18 (75.0) | | 0.274 | |
| Lateral LN | | 23 (44.2) | | 12 (42.9) | | 11 (45.8) | | 0.829 | |
| MRF involvement, n | | 26 (50.0) | | 13 (46.4) | | 13 (54.2) | | 0.578 | |
| NAC, n | |  | |  | |  | | 0.797 | |
| SOX/SOX + bevacizumab | | 27 (51.9) /25 (48.1) | | 15 (53.6) /13 (46.4) | | 12(50.0) /12 (50.0) | |  | |
| NAC completion | | 49 (94.2) | | 27 (96.4) | | 22 (91.7) | | 0.441 | |
| Down staging | | 24 (46.2) | | 14 (50.0) | | 10 (41.7) | | 0.548 | |
| Surgical procedure, n | |  | |  | |  | | 0.937 | |
| LAR/ISR/APR | | 25 (48.1) /13 (25.0) /14 (26.9) | | 14 (50.0)/7 (25.0) /7 (25.0) | | 11 (45.8) /6 (25.0) /7 (29.2) | |  | |
| Tumor size, mm | | 40 (0-80) | | 40 (0-80) | | 38 (5-80) | | 0.875 | |
| UICC 8th edition | |  | |  | |  | |  | |
| T category, n | |  | |  | |  | | 0.019 | |
| pT0-2/T3-4 | | 22 (42.3) /30 (57.7) | | 16 (57.1) /12 (42.9) | | 4 (18.2) /18 (81.8) | |  | |
| N category, n | |  | |  | |  | | 0.006 | |
| pN0/N1-2 | | 32 (61.5) /20 (38.5) | | 22 (78.6)/6 (21.4) | | 10 (41.7) /14 (58.3) | |  | |
| fStage, n | |  | |  | |  | | 0.046 | |
| 0/III/III | | 10 (19.2) /9 (17.3) /13 (25.0)/20 (38.5) | | 7 (25.0) /7 (25.0) /8 (28.6) /6 (21.4) | | 3 (12.5) /2 (8.3) /5 (20.8) /14 (58.3) | |  | |
| Histology, n | |  | |  | |  | | 0.114 | |
| Tubular adenocarcinoma, well differentiated type | | 10 (19.2) | | 3 (10.7) | | 7 (29.2) | |  | |
| Tubular adenocarcinoma, moderately differentiated type | | 31 (59.6) | | 16 (57.1) | | 15 (62.5) | |  | |
| Poorly differentiated type | | 0 | | 0 | | 0 | |  | |
| Mucinous adenocarcinoma | | 1 (1.9) | | 1 (3.6) | | 0 | |  | |
| N.A | | 10 (19.2) | | 8 (28.6) | | 2 (8.3) | |  | |
| Local invasive factor, n | |  | |  | |  | |  | |
| Lymphatic invasion | | 23 (44.2) | | 9 (32.1) | | 7 (29.2) | | 0.143 | |
| Vascular invasion | | 35 (67.3) | | 17 (60.7) | | 18 (75.0) | | 0.274 | |
| Perineural invasion | | 35 (67.3) | | 16 (57.1) | | 19 (79.2) | | 0.091 | |
| R0 resection, n | | 49 (94.2) | | 26 (92.9) | | 23 (95.8) | | 0.559 | |

Sup table 2: Comparison of the clinicopathological characteristics of patients

| Characteristics | All (n=9) | | NAC-effective (n=3) | | NAC-resistant (n=6) | | p value | |
| --- | --- | --- | --- | --- | --- | --- | --- | --- |
| Gender |  | |  | |  | |  | |
| Male, n | 6 (66.7) | | 3 (100.0) | | 3 (50.0) | | 0.238 | |
| Age, year | 62 (47-78) | | 66 (61-78) | | 61 (47-73) | | 0.300 | |
| BMI, kg/m^2^ | 23.5 (18.3-28.0) | | 25.8 (18.3-27.9) | | 22.6 (19.4-28.0) | | 1.000 | |
| CEA, ng/ml | 8.8 (0.9-115) | | 1.9 (0.9-3.6) | | 14.4 (3.8-115) | | 0.020 | |
| UICC 8th edition, n |  | |  | |  | |  | |
| cT category |  | |  | |  | | 0.226 | |
| cT3/T4 | 6 (66.7) /3 (33.3) | | 2 (66.7) /1 (33.3) | | 2 (33.3) / 4(66.7) | |  | |
| cN category |  | |  | |  | | 0.226 | |
| cN0/N1-2 | 3 (33.3) /6 (66.7) | | 2 (66.7) / 1 (33.3) | | 1(16.7) /5 (83.3) | |  | |
| Mesorectal LN | 6 (66.7) | | 1 (33.3) | | 5 (83.3) | | 0.226 | |
| Lateral LN | 4 (44.4) | | 1 (33.3) | | 3 (50.0) | | 0.595 | |
| MRI factor, n |  | |  | |  | |  | |
| EMVI | 9 (100.0) | | 3 (100.0) | | 6 (100.0) | |  | |
| TD | 5 (55.6) | | 0 | | 5 (83.3) | | 0.048 | |
| MRF involvement | 6 (66.7) | | 1 (33.3) | | 5 (83.3) | | 0.226 | |
| NAC, n |  | |  | |  | |  | |
| SOX/ SOX + bevacizumab | 3 (33.3) /6 (66.7) | | 2 (66.7) /1 (33.3) | | 1 (16.7) /5 (83.3) | | 0.226 | |
| NAC completion | 8 (88.9) | | 3 (100) | | 5 (83.3) | | 0.667 | |
| Down staging | 3 (33.3) | | 2 (66.7) | | 1 (16.7) | | 0.226 | |
| Surgical procedure, n |  | |  | |  | | 0.223 | |
| LAR/ISR/APR | 3 (33.3) /3 (33.3) /3 (33.3) | | 1 (33.3) /2 (66.7) /0 | | 2 (33.3) /1(16.7) /3 (50.0) | |  | |
| Tumor size, mm | 45 (13-60) | | 30 (13-55) | | 47 (25-60) | | 0.437 | |
| UICC 8th edition |  | |  | |  | |  | |
| pT category, n |  | |  | |  | | 0.003 | |
| pTis-T2/T3-4 | 3 (33.3) /6 (66.7) | | 3 (100) /0 | | 0/ 6 (100) | |  | |
| pN category, n |  | |  | |  | | 0.012 | |
| pN0/N1-2 | 3 (33.3) /6 (66.7) | | 3 (100) /0 | | 0 /6 (100) | |  | |
| fStage, n |  | |  | |  | | 0.011 | |
| 0/I/II/III | 2 (22.2) /1 (11.1) /0/6 (66.7) | | 2 (66.7) /1 (33.3) /0/0 | | 0/0/0/6 (100) | |  | |
| Histology, n | |  | |  | |  | | 0.134 |
| Tubular adenocarcinoma, well differentiated type | | 3 (33.3) | | 2 (66.6) | | 1 (16.7) | |  |
| Tubular adenocarcinoma, moderately differentiated type | | 6 (66.6) | | 1 (33.3) | | 5 (83.3) | |  |
| Local invasive factor, n |  | |  | |  | |  | |
| Lymphatic invasion | 2 (22.2) | | 0 | | 2 (33.3) | | 0.417 | |
| Vascular invasion | 5 (55.6) | | 0 | | 5 (83.3) | | 0.048 | |
| Perineural invasion | 6 (66.7) | | 0 | | 6 (100) | | 0.012 | |
| pExtramural vascular invasion | 6 (66.7) | | 0 | | 6 (100) | | 0.012 | |
| pExtramural tumor nodule | 5 (55.6) | | 0 | | 5 (83.3) | | 0.048 | |
| R0 resection, n | 8 (88.9) | | 3 (100) | | 5 (83.3) | | 0.667 | |

Sup Table 3: The top 10 enriched pathways from a comparison between NAC-resistant and NAC-effective group

| Rank | Pathway | p value | q value | Count | Proteins |
| --- | --- | --- | --- | --- | --- |
| 1 | Citrate cycle (TCA cycle) | 2.45E-07 | 2.11E-07 | 9 | ACLY/IDH2/IDH3A  SUCLG2/ACO2/DLD  PCK2/PDHB/IDH1 |
| 2 | Valine, leucine and isoleucine degradation | 6.94E-07 | 5.97E-07 | 10 | ACAA2/ACADM/IL4I1  ALDH2/HADH/HIBCH  HMGCS2/DLD/HADHB  ACADS |
| 3 | Carbon metabolism | 5.21E-06 | 4.48E-06 | 13 | IDH2/GOT2/IDH3A  ENO1/HIBCH/SUCLG2  ACO2/GLUD1/ALDOC  DLD/ACADS/PDHB  IDH1 |
| 4 | Sulfur metabolism | 2.02E-05 | 1.73E-05 | 5 | ETHE1/THTR  SELENBP1/SQOR/MPST |
| 5 | Fatty acid degradation | 2.56E-05 | 2.20E-05 | 8 | ACAA2/ACADM/ACSL1  ALDH2/HADH/ACADVL  HADHB/ACADS |
| 6 | Propanoate metabolism | 3.57E-05 | 3.06E-05 | 7 | HIBCH/SUCLG2/LDHA  ECHDC1/DLD/ACADS  MLYCD |
| 7 | Fatty acid metabolism | 1.71E-04 | 1.47E-04 | 8 | ACAA2/ACADM/FASN  ACSL1/HADH/ACADVL  HADHB/ACADS |
| 8 | 2-Oxocarboxylic acid metabolism | 4.12E-04 | 3.54E-04 | 5 | IDH2/GOT2/IDH3A  ACO2/IDH1 |
| 9 | Biosynthesis of amino acids | 1.05E-03 | 9.03E-04 | 8 | IDH2/GOT2/ IDH3A  ENO1/ACO2/ALDOC  MAT2A/IDH1 |
| 10 | Glycolysis / Gluconeogenesis | 3.37E-03 | 2.90E-03 | 7 | ALDH2/ENO1/LDHA  ALDOC/DLD/PCK2  PDHB |

Sup Table 4: The results of OPLS-DA on 5 proteins constituting the sulfur metabolism pathway

| Number | Proteins | p1 | \|p1(corr)\| |
| --- | --- | --- | --- |
| 1 | ETHE1 | -0.0907894 | 0.94813 |
| 2 | SELENBP1 | -0.0799325 | 0.78693 |
| 3 | THTR | -0.0529827 | 0.850015 |
| 4 | SQOR | -0.0238392 | 0.654163 |
| 5 | MPST | -0.026483 | 0.605859 |

Sup Table 5: Clinicopathological characteristics of the SOX NAC patients

| Characteristics | | All (n=57) | |
| --- | --- | --- | --- |
| Gender | |  | |
| Male, n | | 44 (77.2) | |
| Age, year | | 65 (34-77) | |
| BMI, kg/m^2^ | | 21.2 (16.9-30.4) | |
| CEA, ng/ml | | 4.1 (0.8-253) | |
| UICC 8th edition | |  | |
| cT category, n | |  | |
| cT3/T4 | | 47 (82.5) /10 (17.5) | |
| cN category, n | |  | |
| cN0/N1-2 | | 19 (33.3) /38 (66.7) | |
| cStage, n | |  | |
| II/III | | 21 (36.8) /36 (63.2) | |
| NAC completion rate, n | | 52 (91.2) | |
| Down staging, n | | 30 (52.6) | |
| Histological NAC therapeutic effect, n | |  | |
| Grade 1a/1b/2/3 | | 25 (43.9) /15 (26.3) /8 (14.0) /9 (15.8) | |
| Tumor size, mm | | 36 (0-90.0) | |
| UICC 8th edition | |  | |
| pT category, n | |  | |
| pT0-2/T3-4 | | 24 (42.1) /33 (57.9) | |
| pN category, n | |  | |
| pN0/N1-2 | | 36 (63.2) /21 (36.8) | |
| fStage, n | |  | |
| 0/I/II/III | | 10 (17.5) /13 (22.8) /13 (22.8) /21 (36.8) | |
| Histology, n | |  | |
| Tubular adenocarcinoma, well differentiated type | | 13 (22.8) | |
| Tubular adenocarcinoma, moderately differentiated type | | 33 (57.9) | |
| Poorly differentiated type | | 0 | |
| Mucinous adenocarcinoma | | 2 (3.5) | |
| N. A | | 9 (15.8) | |
| Local invasive factor, n | |  | |
| Lymphatic invasion | | 30 (52.6) | |
| Venous invasion | | 41 (71.9) | |
| R0 resection, n | | 55 (96.5) | |
