# Supplementary figures and images for: Exploration of Malignant Characteristics in Neoadjuvant Chemotherapy-Resistant Rectal Cancer, Focusing on Extramural Lesions
Source: Ann Surg Oncol. 2023 Aug 7;30(12):7612–23. doi: 10.1245/s10434-023-13928-z (PMC10562322; doi:10.1245/s10434-023-13928-z)

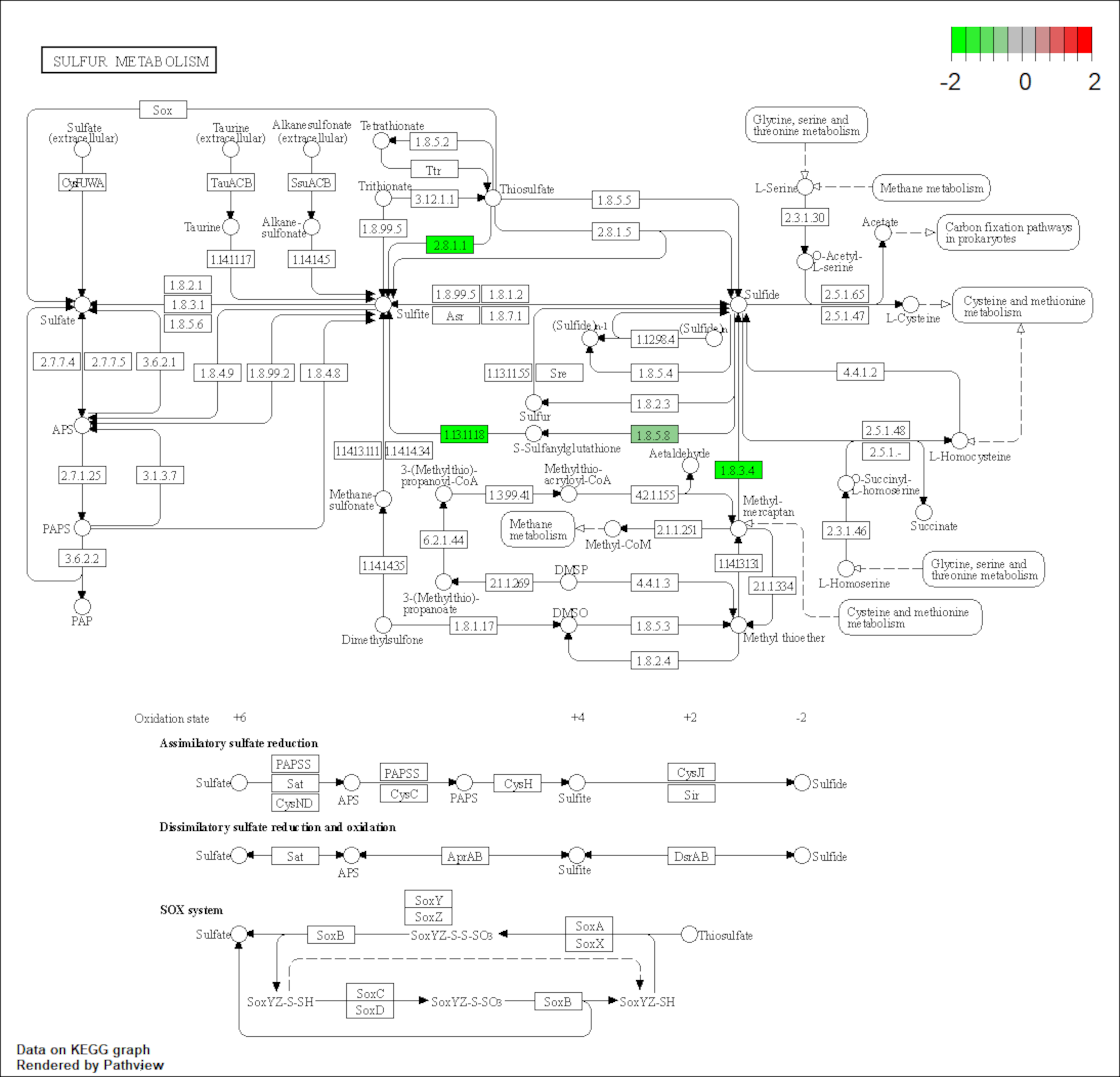

Supplement: Supplementary file 3 — Figure 1. In the KEGG metabolic pathway map for sulfur metabolism (hsa00920), where rectangles and circles represent enzymes and chemical compounds (substrate and products), respectively. EC numbers and gene names are displayed. Molecules with negative (green) fold change represent molecules that were downregulated in the NAC-resistant group. SELENBP1 (1.8.3.4) is directly associated with cysteine and methionine metabolism. (TIF 5294 kb) [file 10434_2023_13928_MOESM3_ESM.tif]
